# Biosorption of Chromium (VI) from Aqueous Solutions onto Fungal Biomass

**DOI:** 10.1155/S1565363304000019

**Published:** 2004

**Authors:** Ismael Acosta R., Xöchitl Rodríguez, Conrado Gutiérrez, Ma. de Guadalupe Moctezuma

**Affiliations:** Autonomous University of San Luis Potosí Faculty of Chemical Sciences (CIEP) Av. Dr. Manuel Nava No. 6. Zona Universitaria San Luis Potosi, S.L.P. C.P. 78320 Mexico

## Abstract

The biosorption of chromium (VI) on eighteen different natural biosorbents: Natural sediment, chitosan, chitin, *Aspergillus flavus* I-V, *Aspergillus fumigatus* I-ll, *Helmintosporium* sp, *Cladosporium* sp, *Mucor rouxii* mutant, *M. rouxii* IM-80, *Mucor* sp-I and 2, *Candida albicans* and *Cryptococcus neoformans* was studied in this work. It was found that the biomass of *C. neoformans*, natural sediment, *Helmintosporium* sp and chitosan was more efficient to remove chromium (VI) (determined spectrophotometrically at 540 nm using diphenylcarbazide as the complexing agent) achieving the. following percentage of removals: 98%, 98% and 63%, respectively. The highest adsorption was obtained with *C. neoformans* and *Helmintosporium* sp at pH 2.0 and 4.0 + 0.2, respectively, at 28^∘^C after 24 hours of incubation, with 0.2 mg/L of cellular biomass.


### Biosorption of Chromium (VI) from Aqueous Solutions onto Fungal Biomass

*Ismael Acosta R., Xöchitl Rodríguez, Conrado Gutiérrez and Ma. de Guadalupe Moctezuma.

*Autonomous University of San Luis Potosi. Faculty of Chemical Sciences (CIEP),*
*Av. Dr. Manuel Nava No. 6. Zona Universitaria. C.P. 78320. San Luis Potosí, S.L.P. México .*
*Tel. 014448262440, Fax: 014448262372. E-mail iacosta@uaslp.mx.*

### ABSTRACT

The biosorption of chromium (VI) on eighteen different natural biosorbents: Natural sediment, chitosan, chitin, *Aspergillus flavus* I-V, *Aspergillus fumigatus* I-II, *Helmintosporium* sp, *Cladosporium* sp, *Mucor rouxii* mutant, *M. rouxii* IM-80, *Mucor* sp-1 and 2, *Candida albicans* and *Cryptococcus neoformans* was studied in this work. It was found that the biomass of *C. neoformans*, natural sediment, *Helmintosporium* sp and chitosan was more efficient to remove chromium (VI) (determined spectrophotometrically at 540 nm using diphenylcarbazide as the complexing agent) achieving the following percentage of removals: 98%, 98% and 63%, respectively. The highest adsorption was obtained with *C. neoformans* and *Helmintosporium* sp at pH= 2.0 and 4.0 + / - 0.2, respectively, at 28°C after 24 hours of incubation, with 0.2 mg/L of cellular biomass.

### INTRODUCTION

The presence of trivalent and hexavalent chromium in the environment can cause many well documented toxic effects. The maximum level of trivalent chromium permitted in wastewater is 5 mg/ml; however, trivalent chromium apparently plays an essential role in plant and animal metabolism when present at low levels. Hexavalent chromium pollution can be due to mining, leather tanning and cement industries, the use in dyes, electroplating, production of steel and other metal alloys, photographic material and corrosive paints. The chromium compounds most severely responsible for environmental pollution are the trivalent compounds, chromium oxide and chromium sulfate and the hexavalent compounds, chromium trioxide, chromic acid and dichromate /1/. The principal techniques for recovering or removing chromium (VI) (which exist in solution as $CrO_4^{2-}$) /2/, from wastewater are: chemical reduction and precipitation, adsorption and ion exchange /3,4/. Currently, the most commonly employed process is reduction of hexavalent chromium to the trivalent form by the addition of a reducing agent, followed by the precipitation as $Cr(OH)_3$ by addition of a basic solution, usually lime, in a basic medium /5/. The objective of this work was to study the biosorption of chromium (VI) on eighteen different natural biosorbents: Natural sediment, the polysaccharides chitosan and chitin, and fifteen biomasses from different fungi.

### EXPERIMENTAL

### Biosorbents

The biosorbents utilized were: Natural sediment, obtained from a lagoon of industrial wastewater in San Luis Potosi, S.L.P., Mexico; the polysaccharides chitosan and chitin (Sigma Chemical Co.); the fungal biomass of *Aspergillus flavus* I-V and *Aspergillus fumigatus* I-II isolated from a mining waste in Zimapan, Hgo, Mexico; *Helmintosporium* sp, *Cladosporium* sp, *Mucor* sp-1 and 2 resistant to zinc, lead and copper isolated from the air collected near a Zinc smelting plant in San Luis Potosí, S.L.P., Mexico; *Mucor rouxii* mutant resistant to copper and lead, obtained by mutagenesis with ethylmethansulfonate; *Mucor rouxii* IM-80 (Wild type); *Candida albicans* and *Cryptococcus neoformans* isolated from a leather works, located in Leon, Gto, Mexico /6,7/.

### Microorganisms and chromium (VI) solutions.

The fungi were grown at 28°C in an agitated and aerated liquid media containing thioglycolate broth, 8 g/L. After 4-5 days of incubation for *A. flavus* I-V, *A. fumigatus* I-II, *Helmintosporium* sp, *Cladosporium* sp, *Mucor* sp 1-2, *M. rouxii* mutant, *M. rouxii*, IM80, *C. albicans* and *C. neoformans*, the cells were centrifuged at 3000 rpm for 5 min, washed twice with trideionized water and then dried at 80°C for 2 h in an oven. The natural sediment was dried at 80°C for 20 min. The polysaccharides were initially washed with EDTA (5%w/v) for one week in constant agitation, changing the EDTA solution every 24 hours; then they were centrifuged at 3000 rpm for 5 min, washed twice with trideionized water and then dried at 80°C for 2 h in an oven.

Chromium (VI) solutions were prepared by diluting 71.86 mg/L stock metal ion solution, which was obtained by dissolving exact quantities of potassium dichromate in distilled water. The concentration range of chromium (VI) solutions was 0.2-0.8 mg/L/ 200 mL. The pH of each solution was adjusted to the required value by adding 1 M $H_2SO_4$ solution before mixing with the microorganism.

### Biosorption studies

A known quantity of dried microorganism was mixed with a known concentration of metal-bearing solution in an Erlenmeyer flask at the desired temperature and pH. The flasks were agitated on a shaker for 4-24 h. Samples of 6 ml were taken in intervals and centrifuged at 3000 rpm for 5 min. The supernatant liquid was separated and analyzed for chromium (VI) ions.

### Determination of chromium (VI) concentration

The concentration of chromium ions in solution was determined spectrophotometrically at 540 nm using diphenylcarbazide as the complexing agent. The sample (5 mL) containing between 0.154 to 0.616 mg/L of chromium (VI) was mixed with 0.5 ml of 1.1 (v/v) $H_2SO_4$, 0.1mL of 85% (v/v) phosphoric acid and 1 mL of 5-diphenylcarbazide in absolute ethanol. After 10 min, the pink-violet colored solution was analyzed for chromium (VI) ions /8/.

### RESULTS AND DISCUSSION

The effect of incubation time, initial pH, temperature and initial metal ion concentration on the adsorption of chromium (VI) onto the microorganisms was investigated. The results are reported as the percentage of removal. Regarding the incubation time, it was found that the highest biosorption occurred at 24 h of incubation for both *C. neoformans* (98%, 0.784 mg/L) and *Helmintosporium* sp (65%, 560 mg/L), since at 48 h the amount of chromium (VI) biosorbed was the same as 24 h (Fig. 1), and these results resemble those reported with solutions by pirite fines /1/. The differences in the incubation time may be explained by unknown changes in the permeability of the fungal cell wall, providing more o less exposition to amino groups of chitosan, in both biomasses analyzed.

The mass of chromium (VI) biosorbed on all microorganisms increased as the initial pH of the adsorption medium decreased. Maximum adsorption capacities were found at pH 2.0 for *C. neoformans* and 4.0 for *Helmintosporium* sp, with a percentage of removal of 98% and 65%, respectively (Fig. 2), which is probably the result of the nature of the chemical interaction of chromium ions with fungi cells. The optimum pH for chromium (VI) removal by peat-moss /9/, seaweed /10/, the biomass of green algae *Chlorella vulgaris* and *Clodophara crispata*, and the fungal biomass of *Rhizopus arrhizus* and *Saccharomyces cerevisiae* was reported to lie in the pH range 1.5-2.5 /11/. Moreover, some studies revealed that although some chromium (VI) was taken up by the biomass, considerable quantities of chromium (VI) were reduced to chromium (III).

Although the biosorption generally occurred al low temperatures, maximum adsorption capacities for chromium (VI) ion on to *C. neoformans* and *Helmintosporium* sp were observed at 28°C (Fig. 3). This is different to the report /11/, which showed that optimum temperature for adsorption to *Zoogloe ramigera* (an activated sludge bacterium), *C. crispata*, and *S. cerevisiae* was at 25°C, and for *R. arrhizus* at 35°C.

The mass of metal ions adsorbed to biomass increased with increasing metal ion concentration in different microorganisms like *Chlorella vulgaris* /11/. Adsorption yields determined at different initial chromium (VI) concentration for both *C. neoformans* and *Helmintosporium* sp, are compared in Fig. 4. For both microorganisms, higher biosorption capacities were observed at low concentrations of metal ions (0.2 mg/L). These concentrations are near the threshold concentration of chromium (VI) in wastewater of the valley of Leon, Mexico /12/, and these data are similar to those shown for *R. arrhizus* and *C. vulgaris* /11/, and *C. maltosa* /13/.

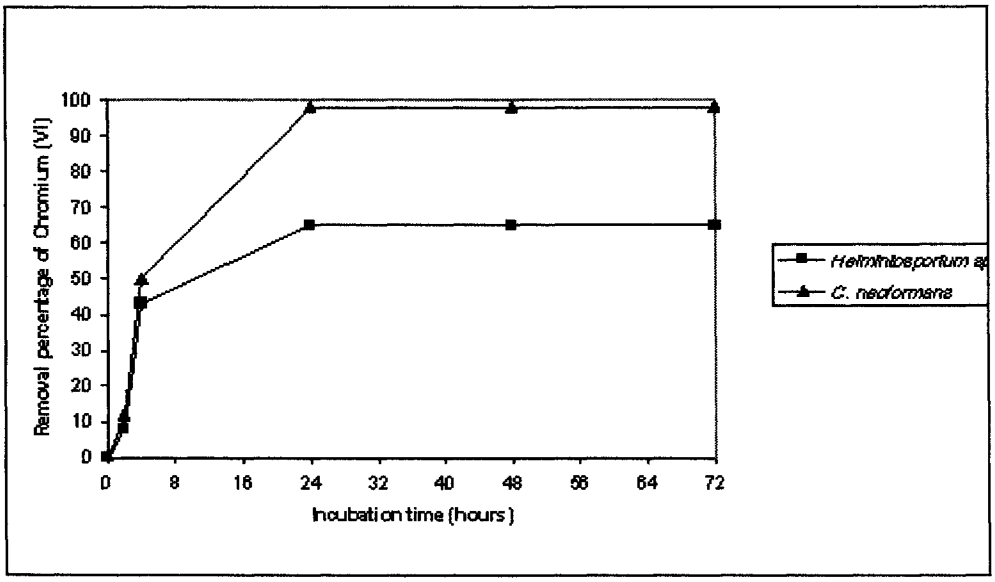

**Fig. 1:** The effect of incubation time on the biosorption of Chromium (VI). 0.2 mg/L, 80 mg biomass, 28°C with constant agitation.

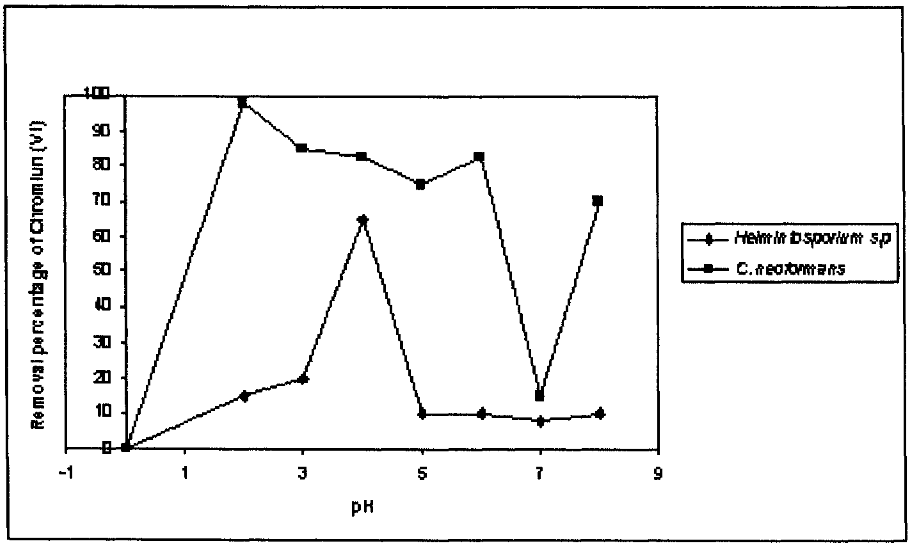

**Fig. 2:** The effect of pH on the biosorption of Chromium (VI). 0.2mg/L/100mL/80mg biomass, 28°C, 24 h of incubation with constant agitation.

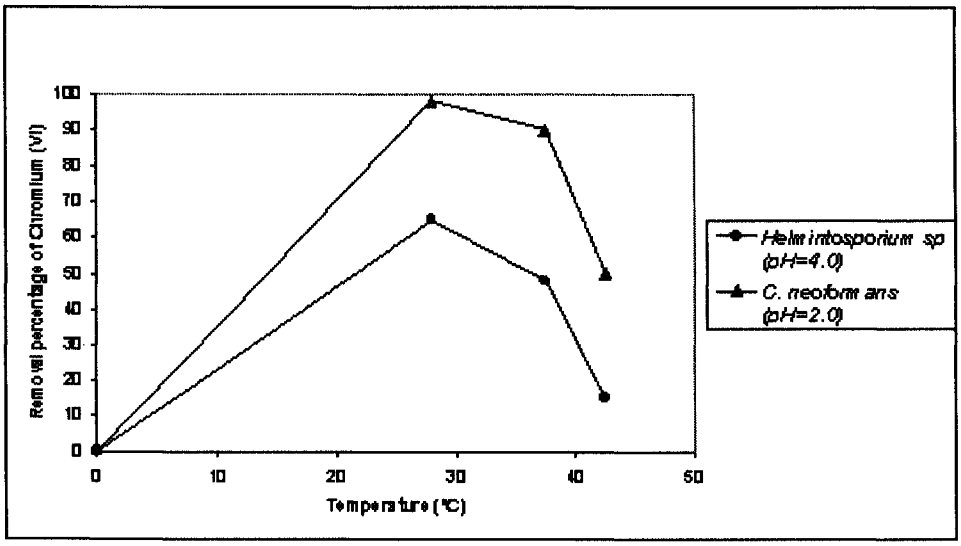

**Fig. 3:** The effect of the incubation temperature on the biosorption on chromium (VI). 0.2 mg/L, 80mg biomass, 24 h of incubation with constant agitation.

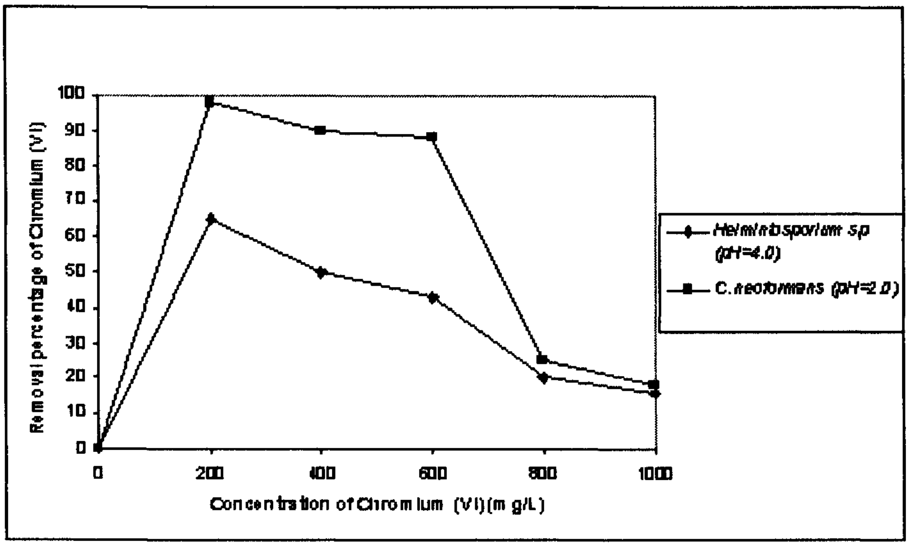

**Fig. 4:** The effect of the concentration of chromium (VI) in solution on the biosorption 80 mg biomass/ 100mL , 28°C, 24 h of incubation with constant agitation.

In Table I, we show the biosorption of chromium (VI) by the different biomasses analyzed. It was found that the biomass of the fungi *C. neoformans* and *Helmintosporium* sp, natural sediment and the polysaccharide chitosan were very efficient at removing the metal in solution (98%, 65%, 98% and 63% respectively). We do not know why the fungal biomass of *C. neoformans* and *Helmintosoporium* sp were the most efficient at removing chromium (VI) in solution. However, this difference may be because the polissacharides of the cell wall could provide binding groups including amino, carboxyl groups and the nitrogen and oxygen of the peptide bonds could be accompanied by displacement of protons, dependent in part upon the extent of protonation as determined by the pH /9,14/.

**Table 1.-**

Biosorption of chromium (VI) on different biomasses.

| BIOMASS | PERCENTAGE OF REMOVAL (%) |
|---|---|
| *Cryptococcus neoformans** | *98* |
| Natural sludge | 98 |
| *Helmintosporium sp* | *65* |
| Chitosan | 63 |
| *Aspergillus flavus* V | 53 |
| *Candida albicans* | *48* |
| *Aspergillus fumigatus* I | 47 |
| *Aspergillus fumigatus* II | 33 |
| *Cladosporium* sp | 33 |
| *Mucor rouxii* mutant | 33 |
| *Aspergillus flavus* II | 32.2 |
| *Aspergillus flavus* IV | 30.1 |
| *Aspergillus flavus* I | 28 |
| *Mucor* sp-2 | 27 |
| *Mucor rouxii* IM80 | 22.5 |
| Chitin | 13.1 |
| *Mucor* sp-1 | 10.4 |
| *Aspergillus flavus* III | 7.4 |

0.2 mg/L/100 ml/80 mg of biomass, 28°C, pH 4.0 +/- 0, 24 h of incubation with constant agitation.
* pH= 2.0 +/- 0.2

The results obtained showed that dried fungi cells are good adsorbing media for chromium (VI) ions. At the concentrations studied, most of the microorganisms can be practically used to remove chromium (VI) present in industrial wastewater. *C. neoformans,* natural sediment, *Helmintosporium* and chitosan are much more efficient than the other microorganisms and chitin for removing higher chromium (VI) concentrations.

In conclusion, the application of biosorption in the purification of wastewater offers a high potential for large-scale exploitation. The natural, abundant, and cheap microbial biomass can be successfully used in selective removal of metal ions from aqueous solutions /15/.

### ACKNOWLEDGEMENTS

To Dr. Roberto Leyva and Q.F.B. Alejandro Salazar for corrections and suggestions.

### REFERENCES

1. A.I. Zouboulis, K.A. Kydros and K.A. Matis, Wat. Res, 7, 1755 (1995).

2. F.A. Cotton and G. Wilkinson, *Advanced Inorganic Chemistry.* 4th ed. Chichester, UK: John Wiley & Sons, 1980.

3. R. Leyva Ramos, A. Juárez and R.M. Guerrero, *Wat. Sci. Tech*, **30** (9), 191 (1994).

4. C. Cervantes, J. Campos-García, S. Devars, F. Gutiérrez-Corona., H. Lozatavera, J.C. Torres-Guzmán, and R. Moreno-Sánchez, FEMS Microbiol. Rev, **25**, 335 (2001).

5. J. Campos, M. Martínez-Pacheco and C. Cervantes, *Antonie van Leeuwenhoek* **68**, 203 (1995).

6. B. Carro, M.G. Moctezuma and I. Acosta, *Femisca*, 473 (2002).

7. C. Gutiérrez, M.G. Moctezuma and I. Acosta, *Memorias del VIII Congreso Iberoamericano de Química Inorgánica*, 65 (2001).

8. A.E. Greenberg, L.S. Clesceri and A.D. Eaton, *Standard Methods for the Examination of Water and Wastewater*, 18th ed. American Public Health Association, Washington, D.C. 3.58-3.60, 1992.

9. D.C. Sharma and C.F. Forster, *Wat. Res*, **27**, 1201 (1993).

10. D. Kratochvil and B. Volesky, *TIBTECH*, **16**, 291 (1998).

11. M. Nourbakhsh, Y. Sag, D. Ozer, Z. Aksu, T. Kutsal and A. Caglar, *Process Biochemistry*, **29**, 1 (1994).

12. M.A. Armienta-Hernández and R. Rodríguez-Castillo, *Environ. Health Persp*, **103**, 47 (1995).

13. R. Ramírez-Ramírez, C. Calvo-Méndez, M. Avila–Rodríguez, P. Lappe, M. Ulloa, R. Vázquez-Juárez and J.F. Gutiérrez-Corona, *Antonie van Leeuwenhoek*, (2003). In Press.

14. Z.A. Aksu, Y. Sag. and T. Kutsal, *Environ. Technol*, **11**, 33 (1990).

15. B. Volesky and Z.R. Holan, *Biotechnol. Progress*, **11**, 235 (1995).

